# Gradient Micropillar Array Inspired by Tree Frog for Robust Adhesion on Dry and Wet Surfaces

**DOI:** 10.3390/biomimetics7040209

**Published:** 2022-11-21

**Authors:** Quan Liu, Fandong Meng, Di Tan, Zhekun Shi, Bo Zhu, Kangjian Xiao, Longjian Xue

**Affiliations:** 1 School of Power and Mechanical Engineering, The Institute of Technological Science, Wuhan University, South Donghu Road 8, Wuhan 430072, China; 2 Institute of Special Polymer Research, Institute of Zhejiang University-Quzhou, 78 Jiuhua Roulevard North, Quzhou 324000, China; 3 Institute of Textiles and Clothing, The Hong Kong Polytechnic University, Hung Hom, Kowloon, Hong Kong, China

**Keywords:** bioinspired material, tree frog, gradient, adhesion, durability

## Abstract

The strong adhesion on dry and wet surfaces and the durability of bioinspired hierarchical fibrillar adhesives are critical for their applications. However, the critical design for the strong adhesion normally depends on fine sub-micron structures which could be damaged during repeat usage. Here, we develop a tree frog-inspired gradient composite micropillars array (GP), which not only realizes a 2.3-times dry adhesion and a 5.6-times wet adhesion as compared to the pure polydimethylsiloxane (PDMS) micropillars array (PP), but also shows excellent durability over 200 repeating cycles of attachment/detachment and self-cleaning ability. A GP consists of stiffer tips and softer roots by incorporating gradient dispersed CaCO_3_ nanoparticles in PDMS micropillar stalks. The modulus gradient along the micropillar height facilitates the contact formation and enhances the maximum stress during the detaching. The study here provides a new design strategy for robust adhesives for practical applications in the fields of robotics, electronics, medical engineering, etc.

## 1. Introduction

To maximize the survival in the complex, dynamic natural environments, functional gradient structures have been developed in many creatures [1,2,3,4,5,6,7]. For instance, the microscale setae of the ladybird beetle *Coccinella septempunctata* have a gradient modulus from 7.2 GPa at the root to 1.2 MPa at the tip, endowing a high flexibility at the seta tip to enhance contact formation and a stiff stalk to maintain mechanical stability [6]. The same strategy of elastic modulus gradient has also been found in the hierarchical setae of gecko, which allows the nanoscale setal tip to form good contact with the counterpart surface, generating a strong adhesion of ~100 kPa [3,8]. Inspired by the modulus gradient in the setae of beetle/gecko, Hensel et al. [9] achieved similar adhesions on rough and smooth substrates by two-phase cylindrical pillars that were composed of a stiff stalk and a soft tip layer, which was prepared by sequenced casting. A smaller thickness of the top layer and a flat interface between the two phases are beneficial to the adhesion performances [10]. A gradually decreasing modulus from the pillar base to tip has also been incorporated into slanted micropillars which showed strong and anisotropic lateral friction forces [11].

Tree frogs, which can easily climb on vertical or even inverted dry/wet surfaces, have inspired the design of structured adhesives for dry and wet conditions [12,13,14,15,16,17]. Using a poly(acrylamide)/poly(vinyl alcohol) hydrogel to mimic the hexagonal epithelial cells in the tree frog, direct solid–solid contact has been suggested to play a major contribution to the wet adhesion [15]. Chen et al. [17] investigated the shape of epithelial cells on the toe pad of the tree frog *Polypedates megacephalus* and found the main shape was hexagonal. Inspired by this finding, stronger friction in the corner direction was demonstrated in slimmer polydimethylsiloxane (PDMS) hexagonal pillars. Meanwhile, Iturri et al. [13] showed higher friction forces in an elongated PDMS hexagonal pillar than regular hexagonal patterned or non-structured surfaces with/without water at the contact interface. By mimicking the densely packed and oriented hard keratin nanofibrils in tree frogs [18], Xue et al. [19] developed composite micropatterns that were composed of PDMS micropillars that were embedded with polystyrene nanopillars, showing improved adhesion and friction at the same time. Inspired by the nanoconcave top of epidermal cells on tree frogs’ toe pads, micropillar arrays with micropits [14] and nanoconcaves [20] on top have been designed and showed higher wet adhesion and friction compared to the arrays of micropillars with flat tops.

Meanwhile, it has been found that the keratinized layer on the toe surface has a modulus of 5–15 MPa, but the effective elastic modulus (*E*_eff_) of the tissue beneath the keratinized layer continuously decreases to 4–25 kPa with the increase of depth in the toe pad [21], of which the modulus gradient is opposite as compared with the setae in geckos and beetles. The gradually softened interlayer maintains the integrity of the patterned epithelial cells, increasing the adaptability to surfaces, while the large *E*_eff_ on the surface is helpful for wear resistance [22]. The incorporation of the modulus gradient that is found in tree frogs into the gecko-inspired polydimethylsiloxane (PDMS) micropillar array with T-shape tips resulted in an enhancement of adhesion of 3.6-times [23]. It has been widely demonstrated that the micro- and nanopillar arrays with T-shape tips are the best structure design to gain strong normal adhesion for various materials [24,25]. Surprisingly, introducing the tree frog-inspired modulus gradient can even further enhance the adhesion performance of the T-shape micropillar array [23]. However, the preparation process of T-shape tips is rather complicated and the fine overhang structure in T-shape tips is rather soft and could be easily damaged during the repeating cycles of attachment/detachment, hindering the advance of T-shape adhesives toward practical applications [26]. Therefore, it is highly needed to simplify the design of micro- and nanopillar array adhesives and develop robust adhesives with prominent adhesion abilities and durability.

**Figure 1:**
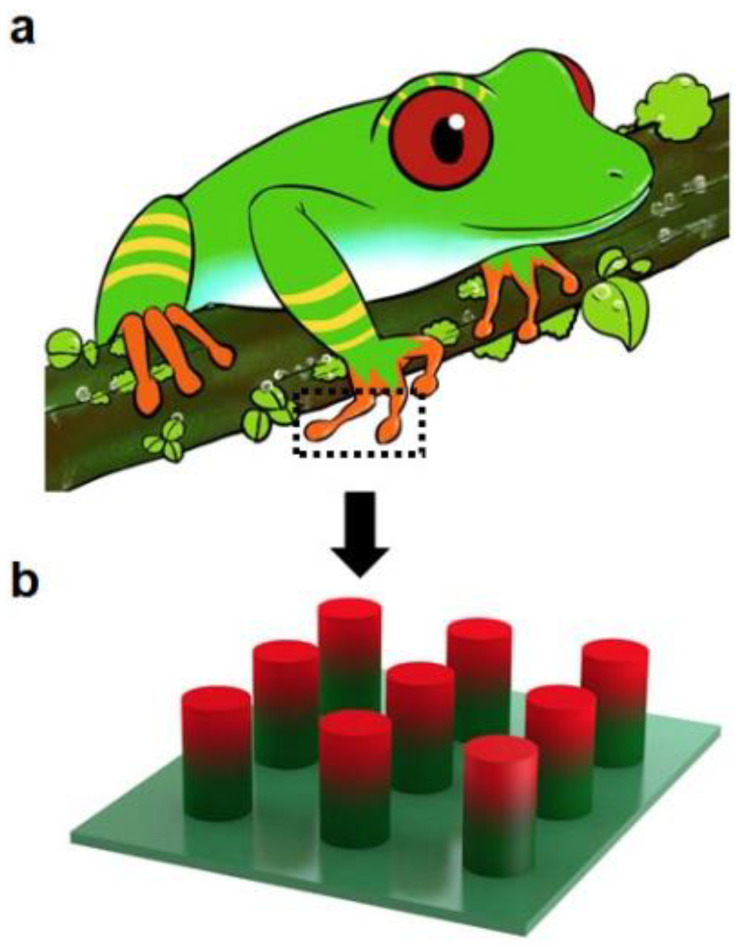
Design principle of GP. (**a**) Schematic of a tree frog. (**b**) Micropillars array inspired by the toe pad of tree frog. Red and green mean relatively larger and smaller elastic modulus, respectively.

Here, we design a gradient composite micropillars array (termed as GP) with a modulus gradually increasing from the micropillar base to tip, mimicking the tree frog’s modulus gradient (Figure 1). The GP presents 2.3-times dry adhesion and 5.6-times wet adhesion as compared to the pure PDMS micropillars array (PP) with excellent durability. The softer base in the GP allows the pillar to adapt to the contacting surface easily, forming reliable contacts. The rigid tip increases the detaching stress and, therefore, enhances the force that is required for the separation. The concept of GPs and the fabrication method can be extended to other material combinations for strong adhesions.

## 2. Experimental Section

### 2.1. Materials

The polydimethylsiloxane (PDMS) elastomer kit (Sylgard 184) was purchased from Dow Corning (Michigan, MI, USA). Polyurethane (PU) resin (ST-1060 A/B) was purchased from BJB Enterprises, Inc., Tustin, CA, USA. CaCO_3_ was purc hased from Suzhou Research Materials Microtech Co., Ltd., Suzhou, China.

### 2.2. Equipment

SU-8 composed of arrays of micropillars were prepared by standard photolithography on lithography machine H94-37 (Sichuan Nanguang Nacuum Technology Co., Ltd., Chengdu, China). To obtain composite micropillars with a gradient distribution of CaCO_3_, centrifugations were carried out using an Eppendorf centrifuge 5810R, Germany. Surface microstructures were visualized by field emission scanning electron macroscopic (MIRA 3 LMH, Tescan AG, Brno, Czech Republic), a Nikon ECLIPSE Ci-L macroscopic, and 3D optical surface profiler (NewView 9000, ZYGO Corp., Middlefield, CT, USA). The elementary analyses were conducted by an energy-dispersive spectroscope (EDS) (X-Max 20, Aztec Energy, Oxford, England). The modulus of composite micropillars was tested on a Hysitron TriboIndenter system (Ti950, Hysitron Inc., Eden Prairie, MN, USA). Macroscopic adhesion was tested by a universal testing machine (Suns Tech UTM2103, Shenzhen, China). Microscopic adhesion tests were carried out on a home-made device with a 5 mm glass sphere as the probe.

### 2.3. Fabrication of Gradient Micropillars (GP)

**Figure 2:**
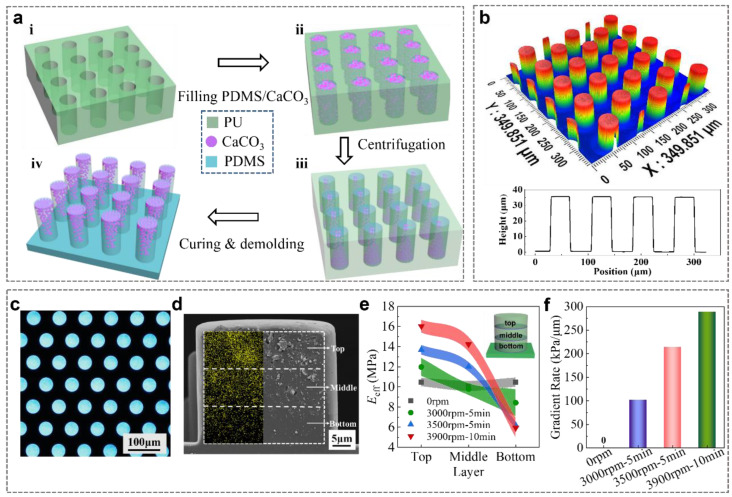
Construction of the GP. (**a**) Schematic of the processing steps for the fabrication of GP: **i** PU mold with patterned cavities; **ii** filling the mold cavities with PDMS/CaCO_3_ composite and scraping the residual mixtures away; **iii** redistribution of the nanoparticles to fabricate gradient composite pillars by centrifugation; **iv** curing and demolding. (**b**) 3D image and height profile of GP array. (**c**) Optical morphology of GP. (**d**) SEM image and EDS map of the cross section of GP. The yellow dots show the distribution of calcium elements (the indication of CaCO_3_). The micropillar is evenly divided into three layers along the direction of micropillar height. (**e**) *E*_eff_ of each layer in GP_70wt%_ under different centrifugal parameters. The inset shows the micropillar is evenly divided into three layers along the direction of micropillar height. (**f**) The gradient rate of GP_70wt%_ under different centrifugal parameters. Shaded regions in (**e**,**f**) indicate the standard deviation.

GP was fabricated by a precise mold replication with the combination of gradient formation in the micropillars by actuation of centrifugal force in Figure 2a. Soft polyurethane (PU) molds containing patterned cylindrical cavities were replicated from SU-8 lithographic templates as previously reported (Figure 2(ai)) [20]. PDMS precursor that was mixed with CaCO_3_ NPs was filled into the PU mold by a vacuum-assisted capillary filling process, and the redundant PDMS/CaCO_3_-precursor composite on the PU mold was scraped away with a spatula (Figure 2(aii)). The PU mold that was filled with PDMS/CaCO_3_ viscous composites was then placed on a plastic Petri dish and transferred onto a swing-bucket centrifuge rotor, followed by the centrifugation at a predefined speed for a given period (Figure 2(aiii)). This centrifugation could generate a horizontal force along the axial direction of the cavities, which forces the CaCO_3_ NPs moving towards the bottom of the cavities in the PU mold and obtain a gradient distribution. The PDMS precursor was cast on the PU mold to create a 500 μm thick backing layer. The whole assembly was cured at 90 °C for 1 h. After the careful demolding from PU mold, the GP was ready (Figure 2(aiv)).

### 2.4. Fabrication of Homogeneous Composite Micropillars Array (HP)

HP was fabricated with the same procedures as GP, but without the centrifugation process.

### 2.5. Fabrication of Pure PDMS Micropillars Array (PP)

PP was fabricated by pouring pure PDMS precursor onto PU mold and cured at 90 °C for 1 h.

### 2.6. Nanoindentation

The modulus of PDMS/CaCO_3_ composite films was performed to calibrate the dependence of *E*_eff_ on *c*_cal_ by quasi-static nanoindentation tests. Force-controlled nanoindentation tests with a maximum loading force of 100 μN were implemented in a Hysitron TriboIndenter system using a standard Berkovich probe (tip radius of ~100 nm) with a rate of 50 nm/s. For the PDMS/CaCO_3_ composite films, the tests were repeated (n = 5) at randomly selected spots on the surface. Elastic modulus values were obtained from the indentation load–depth curves based on the Oliver–Pharr method.

### 2.7. Adhesion Measurement

Macroscopic dry adhesion was tested by a universal testing machine. The substrate of the samples (6 × 6 mm) was fixed onto a smooth glass surface with the micropillar-array side facing upwards. A smooth metal plate was then placed on the micropillar array, and the metal plate formed full contact with the micropillar array under the gravity of the metal plate. The plate was separated from the array at a speed of 50 μm/s, and the maximum pull-off force was recorded, which was the macroscopic adhesion force. Macroscopic wet adhesion was tested by the same procedures as macroscopic dry adhesion, but with 1 mL of deionized water at the contact interface.

Microscopic dry adhesion performance was measured by a home-made device with a 5 mm glass sphere as the probe. The glass sphere probe approached the micropillars array with a velocity of 30 μm/s. At a certain displacement, the contact forms with the micropillars array. The probe was displaced into the micropillar array until a predefined loading force *F*_L_ was reached. Then, the probe was retracted with the same velocity as it moved towards the micropillar array. The pull-off force *F*_ad_ that represents the normal adhesion force of the micropillars array is the force that is required to separate the probe from the micropillar array. *F*_ad_ has the opposite sign of *F*_L_. The adhesion and friction force were repeated 5 times and the mean value was calculated.

## 3. Results and Discussion

### 3.1. Construction of GP

The GP was fabricated by a soft lithography process [27,28], as shown in Figure 2a, and detailed in the experimental section. Briefly, the PDMS/CaCO_3_ mixture was spread onto the PU mold, and filled into the cavities by a vacuum-assisted capillary process (Figure 2(ai,ii)). CaCO_3_ nanoparticles (NPs) were chosen as the filler based on the following reasons: (1) CaCO_3_ is an abundant mineral, occupying 5% of the earth’s crust [29]; (2) the preparation of CaCO_3_ NPs is simple and the size is controllable; (3) CaCO_3_ NPs often serve as reinforcement to improve the mechanical strength of the polymer matrix [30,31]. Nearly 97% CaCO_3_ NPs possess a diameter less than 1 μm, which allows them to fill into the PU mold easily (Figure S1, Supporting Information). The excess mixture of PDMS/CaCO_3_ on the PU mold was carefully removed. Due to the larger density of CaCO_3_ NPs (2.9 g/cm^3^) than PDMS (1.1 g/cm^3^), the CaCO_3_ NPs were propelled towards the bottom of the cavities in the PU mold by applying a centrifugal force along the axial direction of the cavities (Figure 2(aiii)). The following coating of pure PDMS precursor on the mold and curing at 90 °C for 1 h resulted in the composite micropillars with a gradient-distributed CaCO_3_ NPs, which is termed as GP in the following text (Figure 2a and Figure S2, Supporting Information). For the controls, pure PDMS micropillars array (PP) and PDMS/CaCO_3_ homogeneous composite micropillars array (HP) were also fabricated in the same way, but without the addition of CaCO_3_ NPs or the centrifugation process, respectively. Due to simplicity of the methods, four different initial concentrations of CaCO_3_ NPs in PDMS precursor (*c*_cal_ of 10, 30, 50, and 70 wt%) were used to regulate the *E*_eff_ of GP and HP. For convenience, the initial *c*_cal_ is used to identify the samples, such as HP_10wt%_ and GP_10wt%_, in the following text, although the exact *c*_cal_ in the pillars could be different from the initial ones.

The resulting micropillars possess good physical integrity. The 3D images showed that the resulted GP, PP, and HP are 40 μm in diameter, 35 μm in height, and 80 μm in period (Figure 2b and Figure S3a,b, Supporting Information). Under dark-field illumination, the backing layer in GP (HP) is black, while the micropillars in GP are shining. It suggests the presence of CaCO_3_ NPs in the micropillars but not in the backing layer (Figure 2c). The SEM image clearly shows the presence and the gradually increased content of CaCO_3_ NPs from the base to the tip in the pillars of GP (Figure 2d). In contrast, the homogeneous distribution of CaCO_3_ NPs was found in HP (Figure S3c,e, Supporting Information) and no CaCO_3_ NPs in PP (Figure S3d,f, Supporting Information).

The gradient modulus of micropillars in GP was quantitatively characterized [32] (Figure 2e). In order to conveniently characterize the gradient, the micropillar was evenly divided into top, middle, and bottom layers along the micropillar height and the *c*_cal_ of each layer was calculated based on the atom ratio of calcium to silicon. Clear gradient distributions of CaCO_3_ NPs in the three layers were detected in GPs, while a uniform distribution of CaCO_3_ NPs was found in HP (Figure S4, Supporting Information). Since the modulus of CaCO_3_ is much larger than that of PDMS (26 GPa vs. 2.2 MPa), the layer with the larger *c*_cal_ possesses a larger *E*_eff_ (Figure S5, Supporting Information). Under the centrifugation at 3000 rpm for 5 min, *E*_eff_ of the top layer increased to 12.0 ± 0.9 MPa, while *E*_eff_ of bottom layer was 8.4 ± 1.3 MPa (Figure 2e). Increasing the centrifugation speed to 3900 rpm for 10 min, *E*_eff_ steeply increased to 16.0 ± 0.6 MPa in the top layer and decreased to 5.9 ± 0.1 MPa in the bottom layer, forming a distinct gradient in GP_70wt%_ (Figure 2e). The increase in initial *c*_cal_, centrifugal speed and time increases the *E*_eff_ of the top layer and decreases the *E*_eff_ of the bottom layer. The modulus difference between the top and bottom layers divided by height is defined as the gradient rate (Figure 2f). For HP, the gradient rate is 0. The gradient rate of GP_70wt%_ reached 101.4 kPa/μm under the centrifugation at 3000 rpm for 5 min and increased to 288.5 kPa/μm under 3900 rpm for 10 min. When the initial *c*_cal_ was less than 50 wt%, GP can’t reach the largest gradient rate found in GP_70wt%_ (Figure S6a,b, Supporting Information). However, the largest gradient rate of 387.7 kPa/μm was realized in the GP_50wt%_ under a centrifugation at 3500 rpm for 10 min (Figure S6c, Supporting Information). It is assumed to be the result of a moderate viscosity of the mixture. Therefore, we can precisely regulate the gradient rate of the micropillars by combining *c*_cal_ with the centrifugal parameters to mimic the gradient modulus that is found in tree frog toe pads [21].

### 3.2. Adhesion Performances

**Figure 3:**
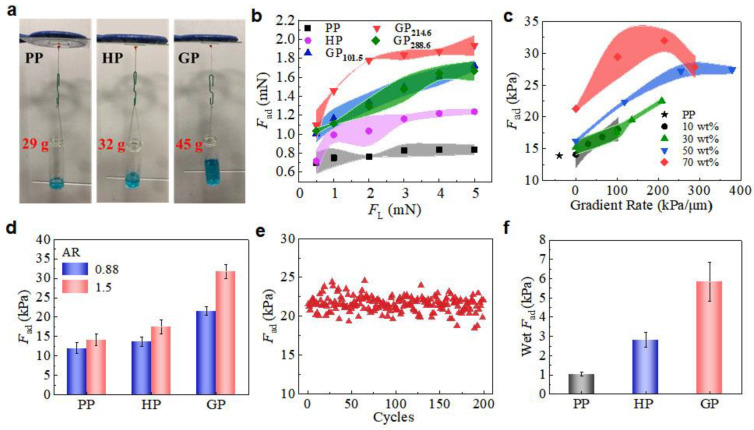
Evaluation of adhesion performances. (**a**) Macroscope adhesion of PP, HP, and GP by hanging weight. (**b**) Dependence of *F*_ad_ of GP_70wt%_ on *F*_L_. (**c**) Dependence of *F*_ad_ of GP_70wt%_ on gradient rate compared with PP. (**d**) Macroscope *F*_ad_ of micropillars with different AR. (**e**) Macroscope *F*_ad_ capacity of 200 attachment/detachment cycles tests for GP. (**f**) Wet *F*_ad_ of GP, HP, and PP. Shaded regions in (**b**,**c**) indicate the standard deviation.

Adhesion performances of micropillars were conducted by macroscopic and microscopic tests. Samples (6 × 6 mm) were finger-pressed onto an upside-down glass surface and weight was hung beneath (Figure 3a). The GP could hold the highest weight of 45 g, much higher that on PP (29 g) and HP (32 g), which clearly suggests the advantage of the gradient modulus of GP in promoting adhesion abilities. Detailed examinations on the dependence of microscopic adhesion force, *F*_ad_, and the loading force (*F*_L_) were carried out on a home-made device with a 5 mm glass sphere as the probe (Figure S7, Supporting Information) [23]. A large *F*_L_ can compensate the possible misalignment between the micropillars and the spherical probe, resulting in a better contact and, therefore, a larger *F*_ad_ [20]. Thus, a higher *F*_ad_ was detected under a larger *F*_L_ (Figure 3b). For instance, the *F*_ad_ of PP slight increased from 0.7 ± 0.1 to 0.8 ± 0.1 mN when *F*_L_ was increased from 0.5 to 5.0 mN. In contrast, the *F*_ad_ of GP_214.6_ (subscript indicates the gradient ratio) greatly increased from 1.1 ± 0.2 to 1.9 ± 0.1 mN, representing a 72.7% increase. The *F*_ad_ of GP_214.6_ under *F*_L_ of 5.0 mN is 122% and 61% higher than PP and HP, respectively. The GP with *c*_cal_ of 10 wt%, 30 wt% and 50 wt% all showed enhancement in *F*_ad_, but the enhancement was less than GP with *c*_cal_ of 70 wt% (Figure S8, Supporting Information). Thus, GP_214.6_ showed not only a much higher adhesion than PP and HP, but also a much stronger dependence on *F*_L_.

The dependence of *F*_ad_ on the gradient rate (under *F*_L_ = 5 mN) was further investigated (Figure 3c). The *F*_ad_ of HP increased from 13.9 ± 0.8 to 21.3 ± 0.4 kPa while *c*_cal_ increased from 0 to 70 wt%, which indicates a positive effect of increasing *E*_eff_ on the *F*_ad_. With *c*_cal_ of 10 wt% and 30 wt%, the *F*_ad_ slightly increased with the increase in gradient rate. At a *c*_cal_ of 50 wt%, the *F*_ad_ increased to 27.2 ± 1.1 kPa at gradient rate of 254.7 kPa/μm. When the initial *c*_cal_ was set to 70 wt%, the *F*_ad_ reached 31.9 ± 1.8 kPa at a gradient rate of 214.6 kPa/μm, which was 2.3-times the PP. The results further confirm that the enhanced adhesion originated from the modulus gradient. The further increase in the gradient rate (for instance, in GP_288.6_), however, reduced the *F*_ad_. The reduction in *F*_ad_ could be the result of the large agglomeration in the pillars (Figure S9, Supporting Information), which may hinder the effective transfer of stress during the detachment. Furthermore, increasing the aspect ratio (AR) of micropillars could enhance the compliant ability of the micropillars to the counterpart surface and, therefore, the adhesion (Figure 3d) [33]. Therefore, the *F*_ad_ of GP_241.6_ was 46.9% improved when AR was increased from 0.88 to 1.50 with the same *c*_cal_ and centrifugal conditions. Interestingly, the adhesion enhancement in GP was also stronger than that of HP (28.1%) and PP (18.2%). As GP has no submicron structures, such as the overhangs in T-shape micropillars, GP_241.6_ has no notable decay in *F*_ad_ after 200 macroscopic cycles test (Figure 3e), suggesting an extraordinary durability of the GP adhesive.

Macroscopic adhesion on a wet surface was also investigated (Figure S10, Supporting Information). As compared to the adhesion on the dry surface, wet *F*_ad_ of GP was much smaller. A wet *F*_ad_ of 5.8 ± 1.0 kPa was detected on GP_214.6_ with deionized water at the contacting interface (Figure 3f). It is reasonable as the captured liquid at the contacting interface hinders the effective formation of contact, reducing the adhesion. On the other hand, however, the wet *F*_ad_ of GP_214.6_ remained the best as compared with PP and HP, which was 5.6- and 2.1-times the PP (1.0 ± 0.1 kPa) and HP (2.8 ± 0.4 kPa), respectively. Once again, it demonstrated the merits of the incorporation of modulus gradient in a micropillar array for adhesion enhancement, in both dry and wet conditions.

**Figure 4:**
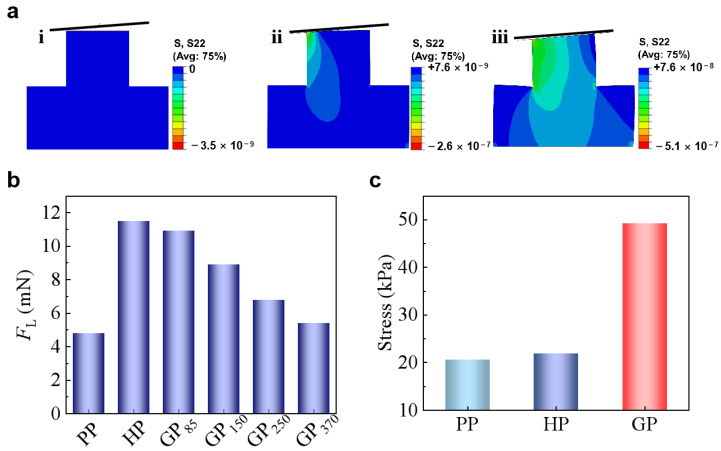
(**a**) Schematic diagrams of the three states of a tilted surface and the micropillar: **i**. before contact, **ii**. during contact, and **iii**. full contact. (**b**) *F*_L_ needed to form full contact with surfaces with tilting angles of 3°. (**c**) Simulated stress at the central separating interface of PP, HP, and GP.

The gradient modulus in GP contributes to the adhesion enhancement based on two mechanisms. Gorb et al. [34] found a narrow neck beneath the contacting tip of the seta of the desert beetle *Dytiscus marginalis* and suggested that the narrow neck could reduce the local bending stiffness, making it easy to adapt to the misaligned surfaces. The soft root in GP could possess a similar function as the narrow neck. To demonstrate the concept, the approaching of a surface with tilting angle of 3° to the GP tip was finite element simulated (Figure 4a). When approached to a tilted surface, the micropillar bends to facilitate the contact formation [23]. While PP has the smallest *E*_eff_ and is the easiest to bend, it needs 4.8 mN to form full contact with the tilted surface (Figure 4b). In contrast, HP needs a much larger *F*_L_ of 11.5 mN to form full contact. GP_370_ needs a *F*_L_ of 5.4 mN to form the full contact, which is quite close to the *F*_L_ that is required for PP. On the other hand, GP with a much larger gradient rate of 85 kPa/μm (GP_85_) could form full contact under a *F*_L_ of 10.9 mN, which is similar to HP. In contrast, although GP also possess a large *E*_eff_ at the free end similar to HP, the soft root of GP increases its flexibility, enhancing the attaching ability to uneven or misaligned surfaces. The larger the gradient ratio, the smaller *F*_L_ is needed for GP to adapt to the tilted surface (Figure 4b).

GP generates a larger stress maximum at the contact interface (*σ*), which is determined by the work of adhesion (*W*) and the effective modulus of the pillar tip (*E*_eff-tip_) [35,36]:

$$\sigma \;=\;\sqrt{\frac{WE_{\mathrm{eff}-\mathrm{tip}}}{A}}$$

where *A* is the area of the pillar end. As the detaching pairs are identical in all the cases here, *W* and *A* are constants. Therefore, the increase of *E*_eff-tip_ would increase the *σ* (Figure 4c), which means a larger force (*F*_ad_) is needed to separate the contacting pair. For instance, GP with *E*_eff-tip_ of 15 MPa has a *σ* of 50 kPa, which is almost 2-times of that in PP and HP. The easier contact formation and the larger stress that is required for detachment, therefore, generate the strongest adhesion on GP as compared with HP and PP.

### 3.3. Applications of GP

**Figure 5:**
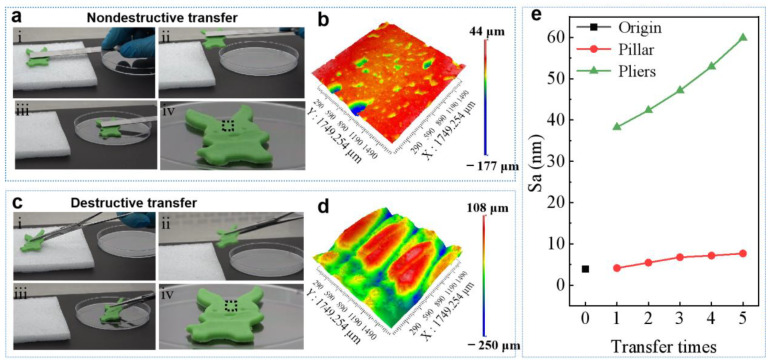
Application of GP adhesion. (**a**) Using a GP grasper to transfer a soft plasticine toy and (**b**) the 3D image, showing the surface of soft object intact as indicate by dashed box in (**a**). (**c**) Using a shape pliers grasper to transfer a soft plasticine toy and (**d**) the 3D image, showing the surface of soft object damaged as indicate by dashed box in (**c**). (**e**) The Sa of the soft plasticine toy after five transfers.

Undesirable damage occurring on the contact surfaces is a common phenomenon when grasping soft objects. In order to avoid this kind of damage, superior adhesion is in great demand on grasping soft objects [37]. Attempts to improve the adhesion performance on soft surfaces have mainly been pursued by the use of special adhesives or octopus-inspired sucker structures [12,37]. Since the soft objects are vulnerable, any excessive external force should be avoided. Therefore, it is highly desirable to develop a reusable adhesive pad with extraordinary adhesion ability with little external force. The strong adhesion of GP can solve this challenge perfectly. A soft plasticine toy with surface roughness (Sa) of 3.91 μm was used to demonstrate the concept (Figure S13, Supporting Information). The excellent adhesion of GP allows it stick to the surface of the soft plasticine toy by applying an ignorable *F*_L_. The higher adhesion under small *F*_L_ makes GP capable of picking up an object with smaller *F*_L_ as compared to PP and HP (Figure 3b). The subsequent peeling at a small angle can release the toy to a designated position easily (Figure 5a). The surface showed no clear deformation as compared to the surface before transportation (Figure 5b). In contrast, although a small force was carefully applied, sharp pliers grasping strongly deformed the surface, leaving bite marks on the surface (Figure 5c,d). After transferred by GP five times, the surface roughness of the soft plasticine toy slightly increased to 7.67 μm (Figure 5e). However, the surface roughness increased significantly to 59.88 μm after the transferring with the sharp pliers grasping five times.

### 3.4. Self-Cleaning Ability of GP

**Figure 6:**
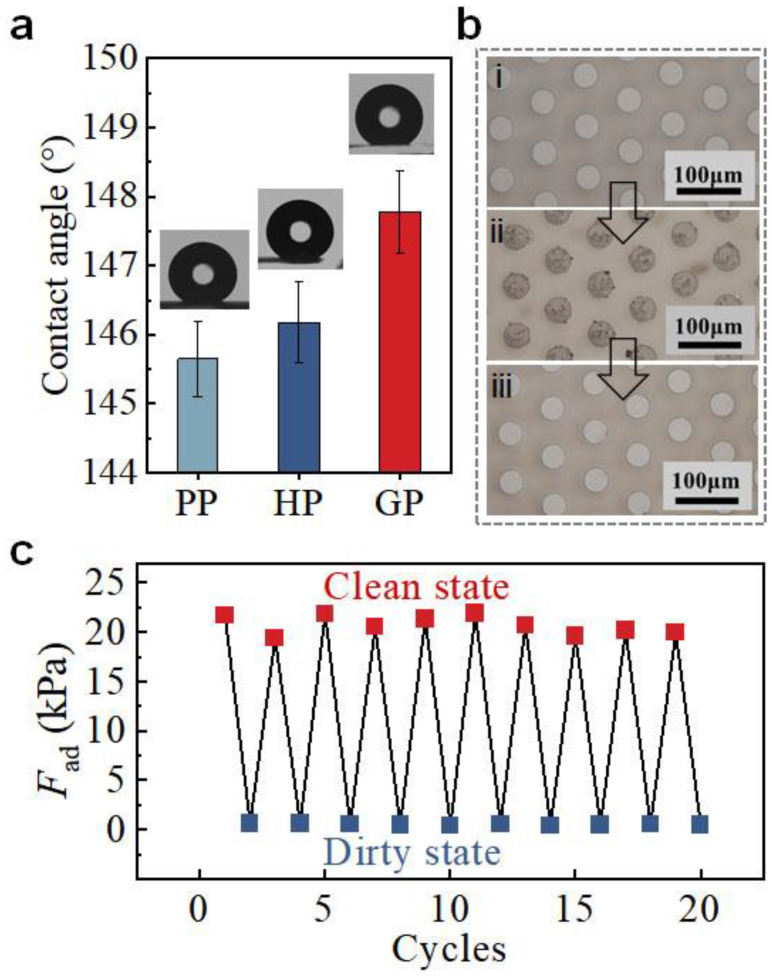
Self-cleaning ability of GP. (**a**) Water contact angles of GP, HP, and PP. (**b**) Photos i to iii represent the initial state of GP, the GP with dust, and the GP after cleaning, respectively. (**c**) *F*_ad_ of GP at states of i, ii, and iii as indicated in (**b**).

The self-cleaning ability is important for the re-usage of GP in dirty environments. The geometry of the micropillar array and the larger roughness on the pillar end of GP (Sa = 28.2 ± 4.9 nm, Figure S12) offer GP a water contact angle of 147.9 ± 0.9°, which is slightly larger than HP and PP (Figure 6a). The good hydrophobicity endows GP with self-cleaning ability [38]. With a simple flushing with water flow, GP can fully recover its adhesion ability after contamination by dust (Figure 6b). After 10 cycles of soiling and cleaning, the adhesion of GP remained unchanged (Figure 6c). It thus undoubtedly demonstrates the robustness of GP in dirty conditions and the ability of self-cleaning, which are of great significance to the reusability application of GP in real environments.

## 4. Conclusions

Inspired by the gradient modulus on the adhesive toe pad of tree frogs, a composite gradient micropillar array (GP) with a gradually increasing elastic modulus from the base to the tip along the micropillar height was successfully designed and constructed. The soft root of GP plays a similar role as the narrow neck structure of the desert beetle, which reduces the bending stiffness of GP and thus facilitates the contact with misaligned surfaces. The high modulus tip increases the maximum stress that is required for detaching, thus enhancing the adhesion. The slightly increased roughness on the pillar top of GP increased the hydrophobicity, which contributes the stronger adhesion in wet conditions and good self-cleaning ability. Thus, GP showed adhesion of 2.3- and 5.6-times compared to the pure PDMS micropillars array under dry and wet conditions, respectively. The results not only provide a robust material with strong dry and wet adhesion, which may find wide applications in various conditions.

## Data Availability

The data presented in this study are available upon request from the corresponding author.

## Supplementary Materials

The following supporting information can be downloaded at: https://www.mdpi.com/article/10.3390/biomimetics7040209/s1, Figure S1: (a) SEM image of CaCO_3_ NPs. (b) The statistics of CaCO_3_ NPs’ diameters; Figure S2: SEM image of GP array; Figure S3: 3D images and height profile of (a) PP and (b) HP. Optical morphology of (c) PP and (d) GP. SEM images of cross sections of (e) PP and (f) HP; Figure S4: (a) Distribution of Ca elements in HP and GP; Figure S5: (a) The dependence of *E*_eff_ on *c*_cal_. *E*_eff_ of each layer in (b) GP_10wt%_, (c) GP_30wt%_ and (d) GP_50wt%_ under different centrifugal parameters; Figure S6: The gradient rate of (a) GP_10wt%_, (b) GP_30wt%_ and (c) GP_50wt%_ under different centrifugal parameters; Figure S7: (a) Home-made device for microscopic adhesion test. (b) Representative force-displacement curve measured on micropillar arrays with loading force (FL) and pull off force (adhesion force, *F*_ad_) indicated; Figure S8: Dependence of *F*_ad_ of (a) GP_10wt%_, (b) GP_30wt%_ and (c) GP_50wt%_ on *F*_L_; Figure S9: SEM images of the cross section of GP_70wt%_ with gradient rate of (a) 214.6 kPa/μm and (b) 288.6 kPa/μm; Figure S10: (a) Macroscopic wet adhesion test by using a universal testing machine. (b) Enlargement image of droplet water at the contact interface; Figure S11: Wet *F*_ad_ of GP with different volume of water in the contact interface; Figure S12: Sa of various micropillars; Figure S13: (a) A photograph image of soft plasticine toy and (b) the 3D image with Sa of 3.91 μm.
