# Supplementary material for: Gradient Micropillar Array Inspired by Tree Frog for Robust Adhesion on Dry and Wet Surfaces
Source: Biomimetics (Basel). 2022 Nov 21;7(4):209. doi: 10.3390/biomimetics7040209 (PMC9680249; doi:10.3390/biomimetics7040209)
Supplement: Supplementary file 1 [file biomimetics-07-00209-s001.zip › biomimetics-2005865-supplementary.pdf]

Supplementary Information

Gradient Micropillar Array Inspired by Tree Frog for Robust Adhesion on Dry and Wet Surfaces

Quan Liu [1,2], Fandong Meng [1], Di Tan [3,*], Zhekun Shi [1], Bo Zhu [1], Kangjian Xiao [1] and Longjian Xue [1,*]

[1] School of Power and Mechanical Engineering, The Institute of Technological Science, Wuhan University, South Donghu Road 8, Wuhan 430072, China
[2] Institute of Special Polymer Research, Institute of Zhejiang University-Quzhou, 78 Jiuhua Roulevard North, Quzhou 324000, China
[3] Institute of Textiles and Clothing, The Hong Kong Polytechnic University, Hung Hom, Kowloon, Hong Kong, China
* Correspondence: di-itc.tan@polyu.edu.hk (D.T.); xuelongjian@whu.edu.cn (L.X.)

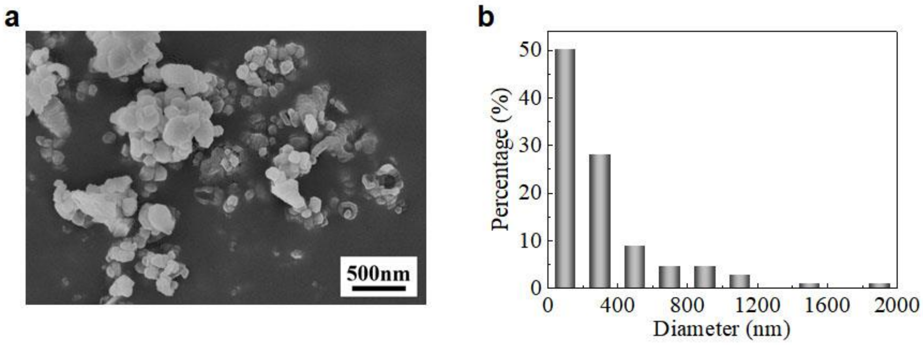

Figure S1. (a) SEM image of $CaCO_3$ NPs. (b) The statistics of $CaCO_3$ NPs' diameters.

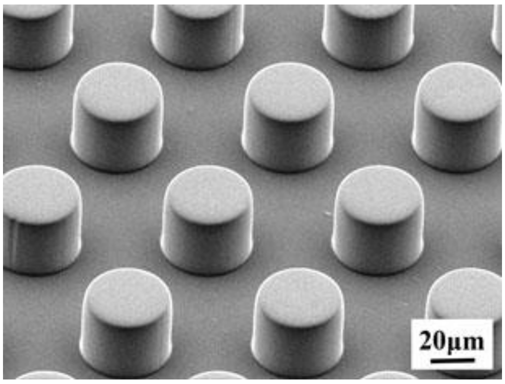

Figure S2. SEM image of GP array.

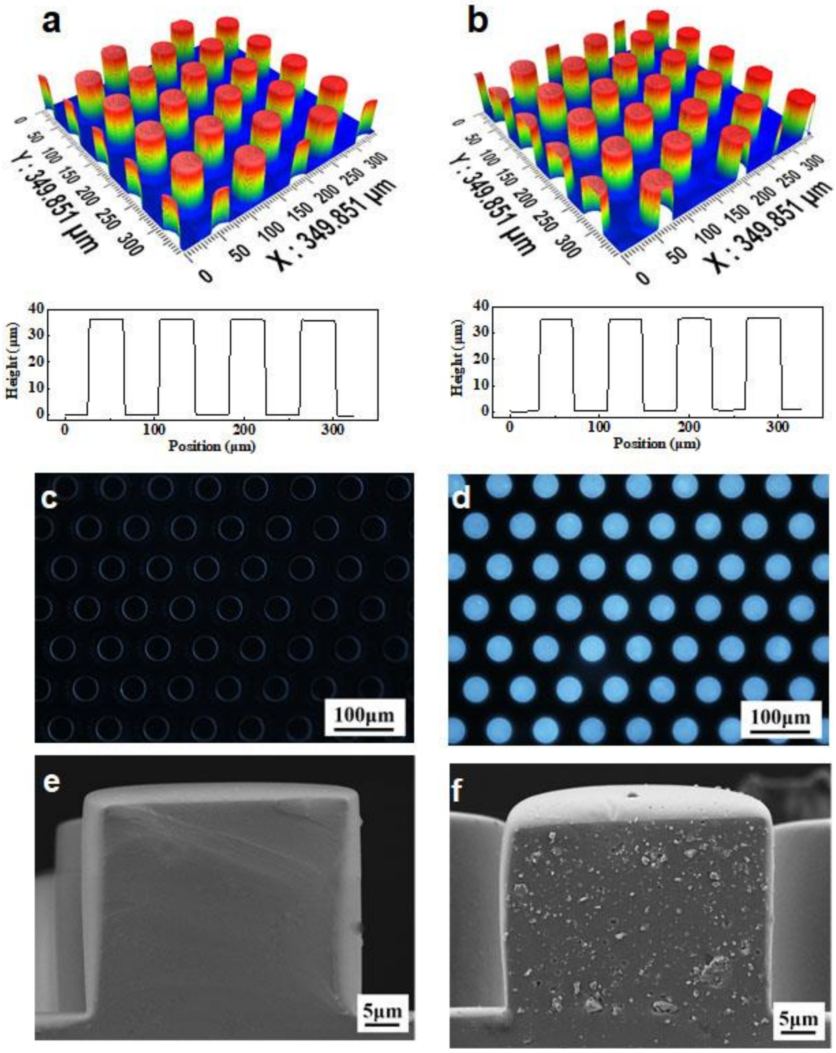

Figure S3. 3D images and height profile of (a) PP and (b) HP. Optical morphology of (c) PP and (d) GP. SEM images of cross sections of (e) PP and (f) HP.

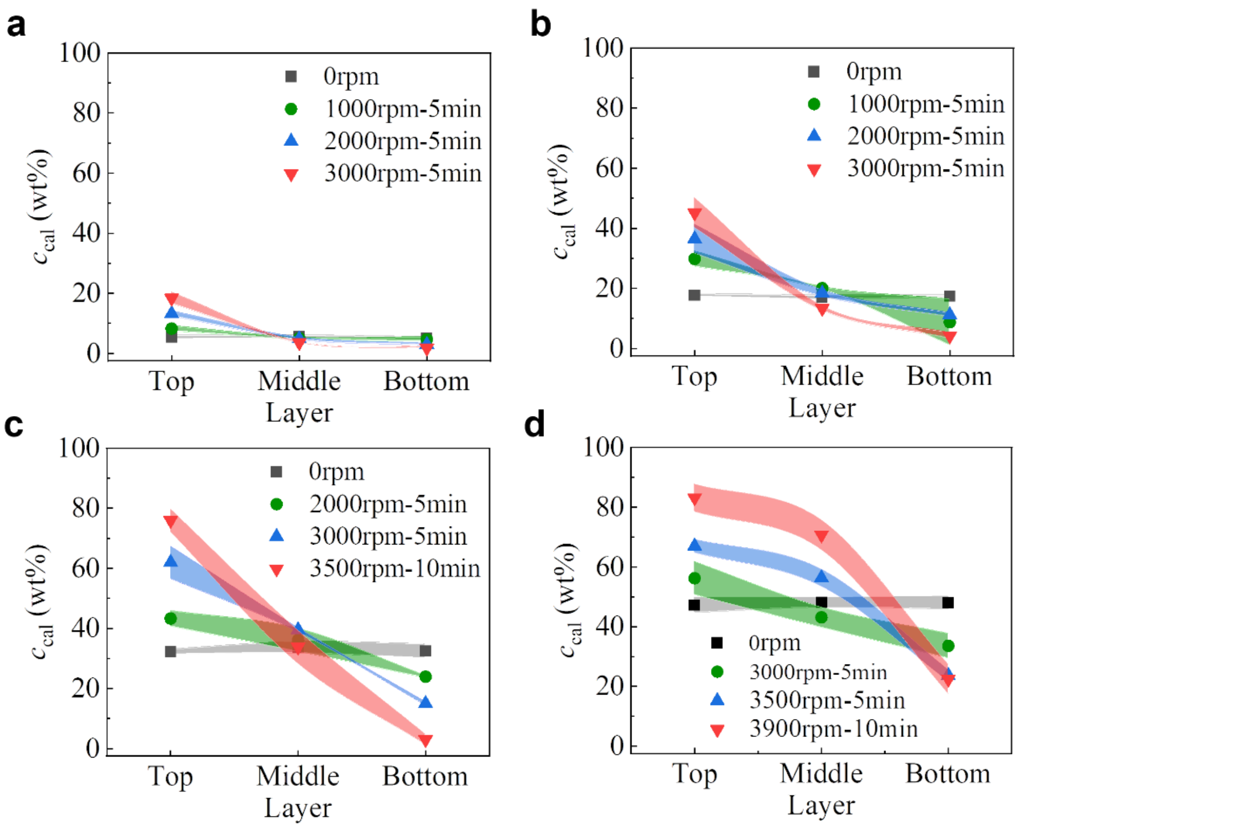

Figure S4. The c_{cal} in different layer of (a) $GP_{10wt\%}$, (b) $GP_{30wt\%}$, (c) $GP_{50wt\%}$ and (d) $GP_{70wt\%}$ under different centrifugal parameters.

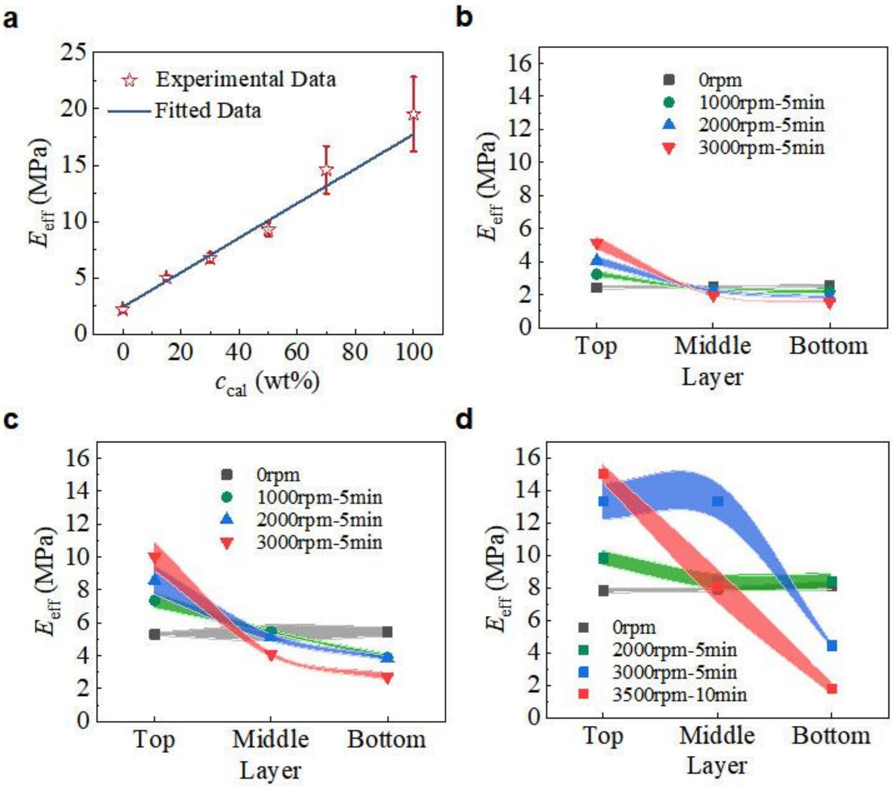

Figure S5. (a) The dependence of E_{eff} on c_{cal}. E_{eff} of each layer in (b) $GP_{10wt\%}$, (c) $GP_{30wt\%}$ and (d) $GP_{50wt\%}$ under different centrifugal parameters.

\

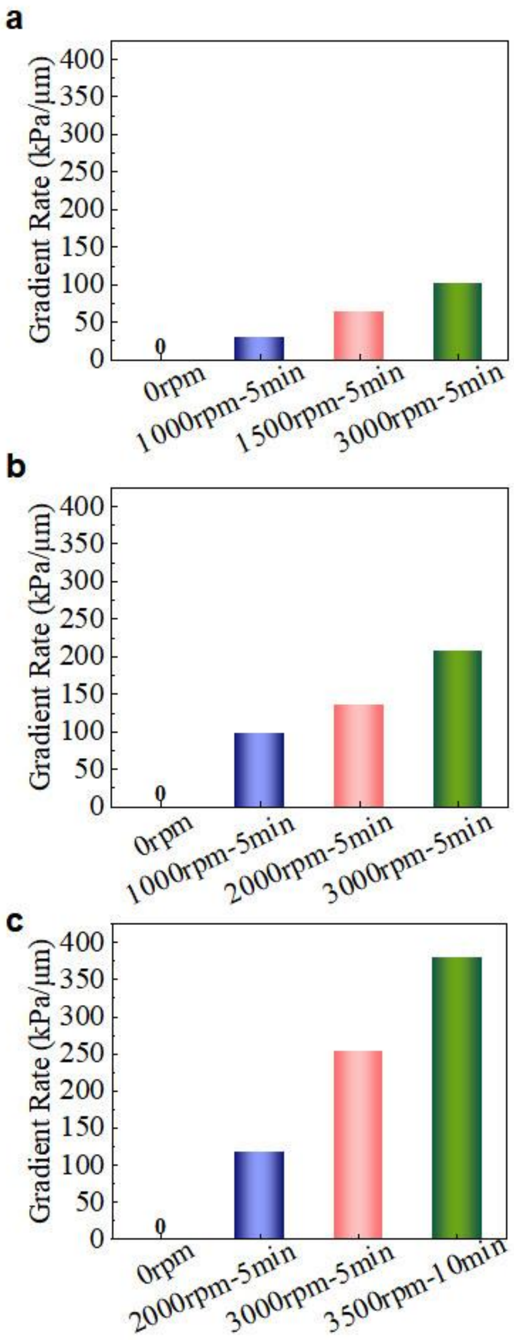

Figure S6. The gradient rate of (a) $GP_{10wt\%}$, (b) $GP_{30wt\%}$ and (c) $GP_{50wt\%}$ under different centrifugal parameters.

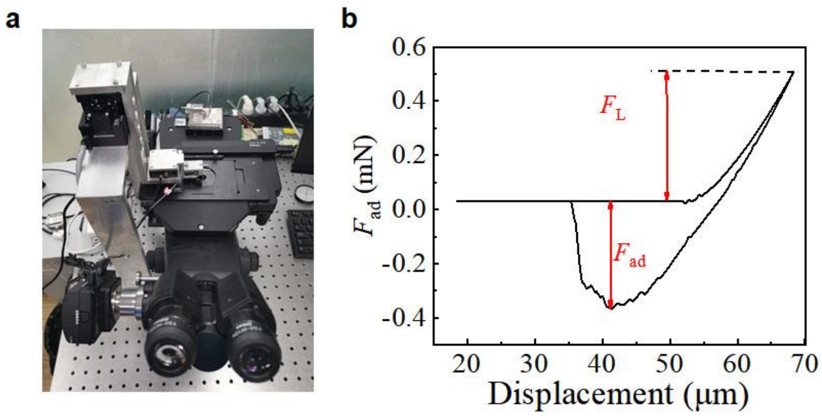

Figure S7. (a) Home-made device for microscopic adhesion test. (b) Representative force-displacement curve measured on micropillar arrays with loading force (F_L) and pull off force (adhesion force, F_{ad}) indicated.

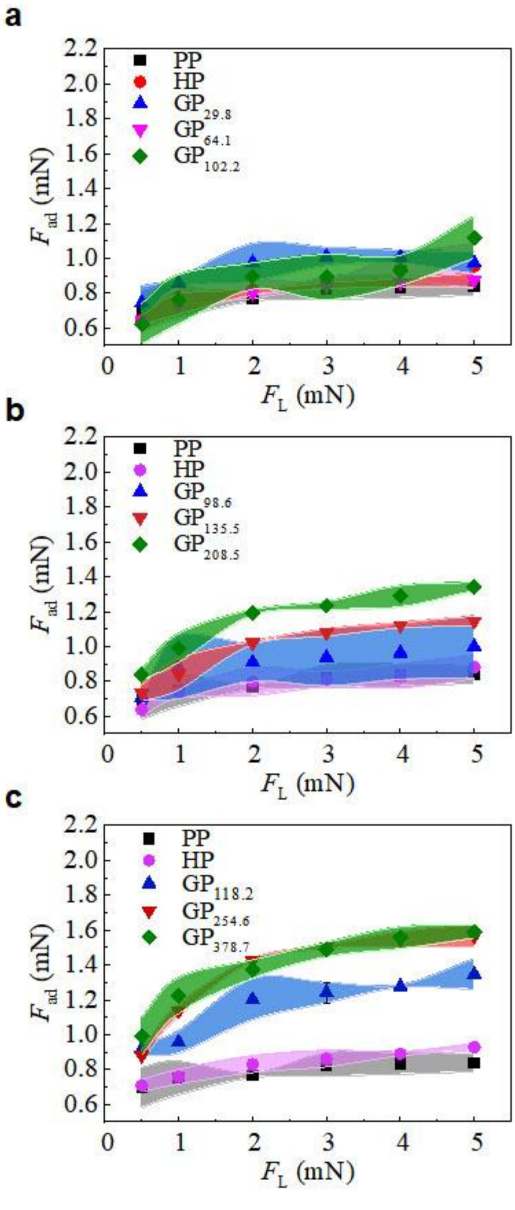

Figure S8. Dependence of F_{ad} of (a) $GP_{10wt\%}$, (b) $GP_{30wt\%}$ and (c) $GP_{50wt\%}$ on F_L.

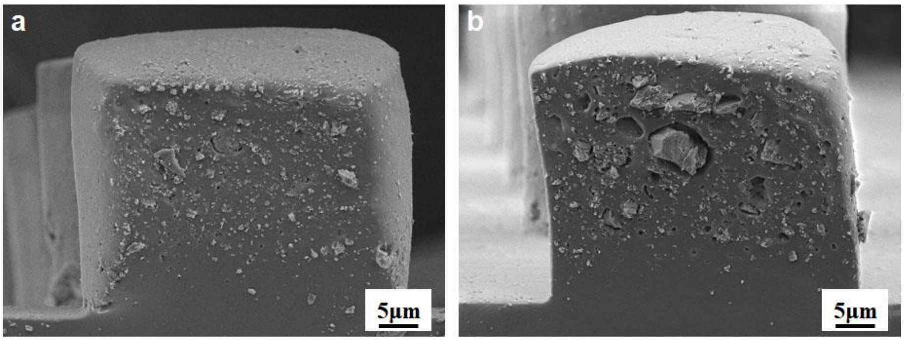

Figure S9. SEM images of the cross section of $GP_{70wt\%}$ with gradient rate of (a) 214.6 kPa/μm and (b) 288.6 kPa/μm.

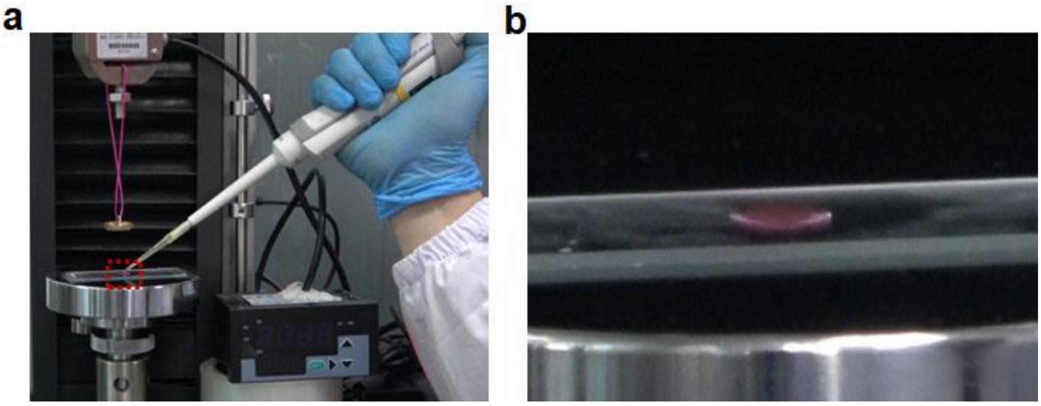

Figure S10. (a) Macroscopic wet adhesion test by using a universal testing machine. (b) Enlargement image of droplet water at the contact interface.

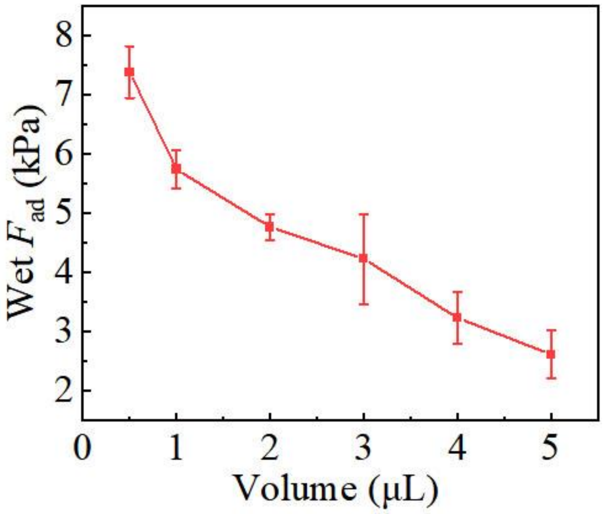

Figure S11. Wet F_{ad} of GP with different volume of water in the contact interface. .

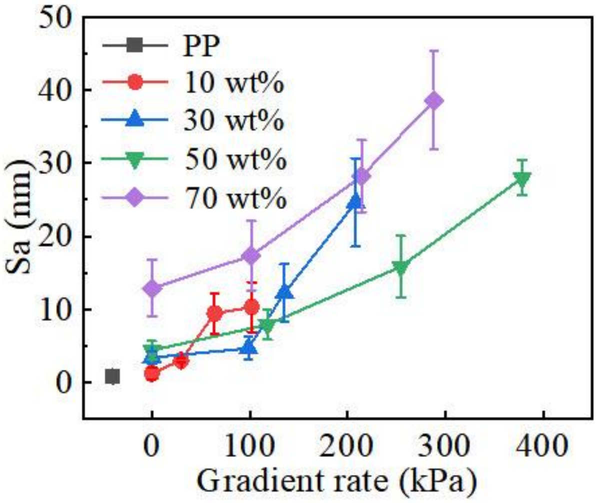

Figure S12. Sa of various micropillars.

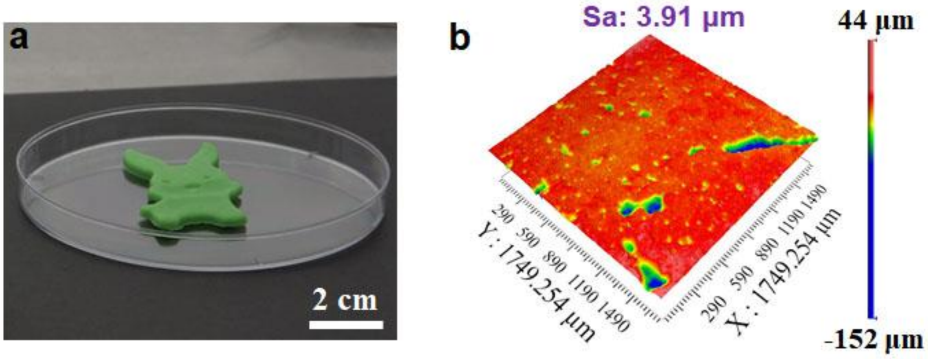

Figure S13. (a) A photograph image of soft plasticine toy and (b) the 3D image with Sa of 3.91 μm.
